# A sporulation-specific, *sigF*-dependent protein, SspA, affects septum positioning in *Streptomyces coelicolor*

**DOI:** 10.1111/mmi.12466

**Published:** 2013-12-19

**Authors:** Angelos Tzanis, Kate A Dalton, Andrew Hesketh, Chris D den Hengst, Mark J Buttner, Annabelle Thibessard, Gabriella H Kelemen

**Affiliations:** 1 University of East Anglia, Norwich Research ParkNorwich, UK; 2 John Innes Centre, Norwich Research ParkNorwich, UK

## Abstract

The RNA polymerase sigma factor SigF controls late development during sporulation in the filamentous bacterium *Streptomyces coelicolor*. The only known SigF-dependent gene identified so far, *SCO5321*, is found in the biosynthetic cluster encoding spore pigment synthesis. Here we identify the first direct target for SigF, the gene *sspA*, encoding a sporulation-specific protein. Bioinformatic analysis suggests that SspA is a secreted lipoprotein with two PepSY signature domains. The *sspA* deletion mutant exhibits irregular sporulation septation and altered spore shape, suggesting that SspA plays a role in septum formation and spore maturation. The fluorescent translational fusion protein SspA–mCherry localized first to septum sites, then subsequently around the surface of the spores. Both SspA protein and *sspA* transcription are absent from the *sigF* null mutant. Moreover, *in vitro* transcription assay confirmed that RNA polymerase holoenzyme containing SigF is sufficient for initiation of transcription from a single *sspA* promoter. In addition, *in vivo* and *in vitro* experiments showed that *sspA* is a direct target of BldD, which functions to repress sporulation genes, including *whiG*, *ftsZ* and *ssgB*, during vegetative growth, co-ordinating their expression during sporulation septation.

## Introduction

*Streptomyces coelicolor* is a multicellular, filamentous bacterium that is a model organism for both bacterial development and antibiotic production. Unlike in most bacteria, cell division in *S. coelicolor* is incomplete during vegetative growth because of the lack of regular divisional septa formation followed by cell–cell separation, which results in long, branched, multigenomic filaments that grow across and into a solid medium. In response to yet unknown signals, aerial filaments break the surface tension and branch away from the network of substrate mycelium (Elliot *et al.*, 2008; McCormick and Flardh, 2012; Swiercz and Elliot, 2012). Formation of aerial hyphae depends on a set of regulatory genes, called the *bld* genes, encoding an RNA polymerase sigma factor BldN, transcription factors, such as BldC and BldD or a rare tRNA, *bldA* (Elliot *et al.*, 2008; Swiercz and Elliot, 2012). In addition to the regulatory genes, the hyphal emergence into the air is facilitated by hydrophobic, cell surface-associated proteins, such as SapB and the ‘chaplins’, all exhibiting surfactant properties (Claessen *et al.*, 2003; Elliot *et al.*, 2003a; Kodani *et al.*, 2004; Willey *et al.*, 2006).

Initiation of sporulation begins with the cessation of hyphal growth exclusively in the aerial hyphae followed by the separation of the tip-proximal sporogenic compartment and the subapical stem compartment (Kwak *et al.*, 2001; Dalton *et al.*, 2007; Flardh and Buttner, 2009). In the sporogenic hyphae, 50–100 sporulation septa are laid down synchronously at ∼ 1 μm intervals to give rise to the unigenomic pre-spore compartments, while the fate of the subapical stem is cell lysis. Sporulation septation depends on the cell division protein FtsZ, which, unlike in most bacteria, is not essential during vegetative growth in *Streptomyces* (McCormick *et al.*, 1994). The increased expression of FtsZ in the aerial hyphae is the result of transcription from a second promoter, which is specific to the aerial hyphae and is dependent on the early *whi* genes (Flardh *et al.*, 2000). Increased levels of FtsZ result in the formation of the initially helical FtsZ filaments followed by FtsZ rings that are spaced evenly along the sporogenic hyphae (Schwedock *et al.*, 1997; Grantcharova *et al.*, 2005). Sporulation septation coincides with chromosome segregation that is governed by the ParA/ParB segregation protein pair (Jakimowicz *et al.*, 2005; 2007,) together with the proteins, Smc, ScpA and ScpB that are involved in chromosome organization (Dedrick *et al.*, 2009; Kois *et al.*, 2009).

While numerous studies focus on the highly co-ordinated event of sporulation septation, much less is known about the process of spore maturation. Transformation of the pre-spore compartments into mature spores includes changes in the cell wall structure by the generation of a thick spore wall, changes in the cell shape from cylindrical to ovoid and finally, changes in chromosome organization producing highly compact DNA in mature spores. Cell shape and cell wall synthesis in the spores rely on the bacterial cytoskeletal proteins, MreB and Mbl (Mazza *et al.*, 2006; Heichlinger *et al.*, 2011). In rod-shaped bacteria, such as *Escherichia coli* or *Bacillus subtilis*, cell wall synthesis of the lateral cell wall is dependent on members of the actin-like MreB family, including MreB, Mbl and MreBH (Cabeen and Jacobs-Wagner, 2010; White and Gober, 2012). In *Streptomyces* the MreB-like proteins are not involved in hyphal growth and their role is exclusive to spore development. Lack of both MreB and Mbl results in deformations in spore shape and the absence of the thick spore wall characteristic of the wild-type, mature spores. This suggests that similar to unicellular rod-shaped bacteria, MreB-like proteins are controlling cell wall synthesis during the transformation of pre-spore compartments to mature spores in *Streptomyces* (Mazza *et al.*, 2006; Heichlinger *et al.*, 2011). We know very little about the enzymes that are involved in the turnover of the spore wall, although as the major component of bacterial cell wall is the three dimensional polymer, peptidoglycan, they are expected to include both peptidoglycan synthases and hydrolases. Recently, a group of cell wall hydrolytic enzymes, SwlA, SwlB and SwlC, have been implicated in spore development (Haiser *et al.*, 2009). Chromosome compaction in the spores relies on nucleoid-associated proteins, such as HupS (Salerno *et al.*, 2009) and the Dps proteins (Facey *et al.*, 2009; 2013;, 2011,).

Spore maturation in the late stages of differentiation is governed by an RNA polymerase sigma factor, σ^F^. Spores of the *sigF* mutant have thinner walls and smaller sizes and the spore chains of the *sigF* mutant do not fragment as easily as those of the wild-type strain, suggesting a deficiency in spore-spore separation (Potuckova *et al.*, 1995; Rezuchova *et al.*, 1997). Within the spores, the chromosome of the *sigF* mutant is not as tightly packed as that of the wild-type strain suggesting that SigF targets will include genes that are involved in spore maturation. Surprisingly, the only SigF target that has been identified to date is the promoter of ORF8 (SCO5321) in the *whiE* biosynthetic cluster for the dark grey spore pigment production (Kelemen *et al.*, 1998; Novakova *et al.*, 2004). While transcription of *SCO5321* was dependent on *sigF*, *in vitro* run-off transcription failed to confirm that SigF was directly involved in initiating transcription from this promoter.

Here we identify a novel spore-specific protein, SspA and we demonstrate that the single promoter of *sspA* is not only dependent on *sigF*, but its transcription is directed by RNA polymerase holoenzyme containing SigF, which establishes *sspA* as the first direct target identified for the sigma factor, SigF.

## Results

### 2D-PAGE of spore extracts identifies SspA as a potential SigF target

**Fig 1:**
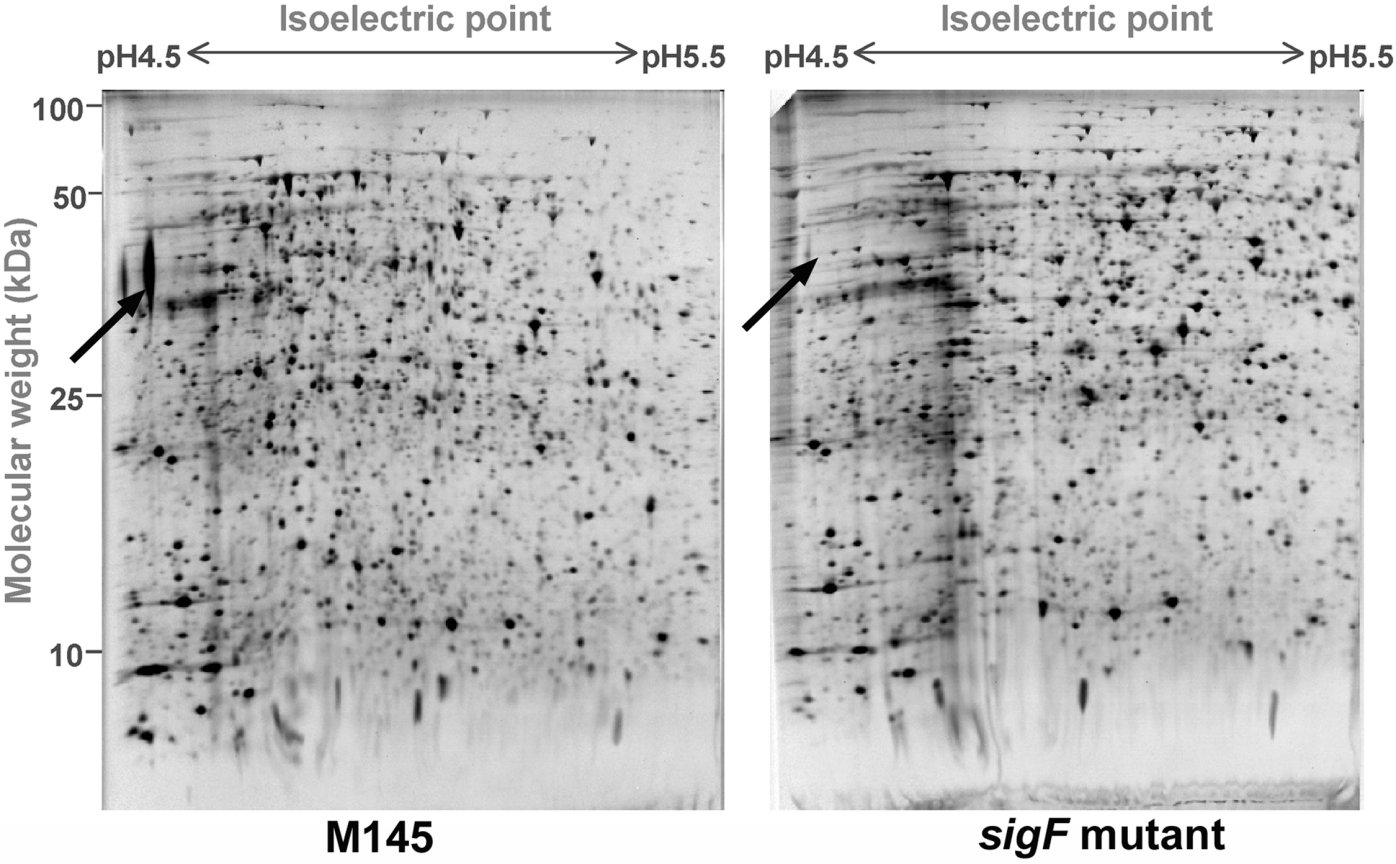
2D-PAGE analysis of spore proteins identifies SspA (marked with an arrow) in the wild-type spores but not in the spores of the *sigF* mutant. Details of sample preparation and 2D gel-electrophoresis are described in *Experimental procedures*.

In order to characterize the role of SigF in spore maturation in *S. coelicolor* we compared protein extracts from spores of wild-type, M145 and *sigF* mutant strains using two-dimensional polyacrylamide gel-electrophoresis (2D-PAGE). Due to the thick spore wall of *Streptomyces* spores, producing protein extracts from spores is not a trivial matter. Sonication, a method that is often reported when presenting proteomics data on spores, only extracted a minority of proteins from the spore wall in our hands and did not release proteins from inside the spores efficiently and reliably. Instead, a mechanical disruption method in which spores were ground in the presence of glass beads under liquid nitrogen, was used to efficiently extract proteins from spores for further analysis. Comparison of protein extracts from spores of the wild-type and *sigF* mutant strains following separation using a narrow pH-range IPG strip of pH 4.5–5.5 and colloidal Coomassie staining revealed a highly abundant protein in the wild-type extract that was clearly absent in the *sigF* mutant (Fig. 1). MALDI-TOF analysis identified this spot as the protein SCO7434, with 9 matched peptides covering 33% of the database protein sequence and producing a probability-based MOWSE score of 94 (expect value 2.2e-05)*.* We therefore designated this protein as SspA (sporulation-specific protein).

### SspA is a lipoprotein with two PepSY domains

The *sspA* gene (*SCO7434*) of *S. coelicolor* has been annotated as a gene encoding a putative lipoprotein with a possible N-terminal signal sequence and prokaryotic membrane lipoprotein lipid attachment site. Indeed, SignalP4.1 (Petersen *et al.*, 2011) identified the first 32 amino acids (aa) as a signal sequence. Moreover, the conserved lipobox motif of L_−3_-[A/S/T]_−2_-[G/A]_−1_-C_+1_ (LipoP 1.0; Juncker *et al.*, 2003) is present in SspA (LTAC), predicting a lipoprotein signal peptidase cleavage site between amino acids 28 and 29. This suggests that SspA is secreted through the cell membrane and it is attached to the membrane, possibly between the lipid bilayer and the peptidoglycan wall. It is consistent with the appearance of SCO7434 in 2D-PAGE gels (Fig. 1) where this protein formed a ‘smear’ characteristic of lipoproteins, which, during cell lysis, maintain covalent attachment to certain lipids of the cell membrane. In order to gain more information about SspA we performed an iterative blast search (Psi-Blast at NCBI; http://www.ncbi.nlm.nih.gov/BLAST.cgi) together with protein domain identification searches using SMART or Pfam (at http://smart.embl-heidelberg.de and at http://www.sanger.ac.uk). According to the SMART and Pfam searches, the 253 aa long SspA contains two PepSY domains between 81 and 167 aa and 188–249 aa. blast searches identified homologous domains that are widespread among bacteria, often as the N-terminal propeptide domain of the M4 family of peptidases, which contains the thermostable thermolysins and related thermolabile neutral proteases (Yeats *et al.*, 2004). Bioinformatics analysis of the distribution of PepSY domains among bacteria and fungi has indicated three main groups for PepSY containing proteins (Yeats *et al.*, 2004). The first group contains M4 peptidases; members of the second group have normally one to five copies of the PepSY domain and no other domains apart from a signal peptide signature and the third group contains one or two PepSY domains surrounded by transmembrane helices. *S. coelicolor* possesses eight PepSY domain-containing proteins, none of which has been characterized up to date. Four proteins, SCO1226, SCO2474, SO5446 and SCO5447, belong to group 1, carrying M4 peptidase domains. Two proteins, SspA and SCO5402 belong to group 2 with two PepSY domains. Finally, SCO0863 and SCO0987 are group 3 proteins with additional transmembrane helices.

### The *sspA* null mutant has irregular spore size

In order to identify the function of *sspA* in spores, we generated an *sspA* null mutant by gene replacement using PCR targetting (Gust *et al.*, 2003). We replaced the *sspA* gene within the cosmid St6D11 with an apramycin resistance cassette and *oriT* function using lambda Red recombinase (Datsenko and Wanner, 2000; Gust *et al.*, 2003). The mutant cosmid was then introduced into *S. coelicolor* M145 using conjugation, and the apramycin-resistant and kanamycin-sensitive colonies were selected and screened for, in order to generate the *sspA* null mutant, designated K55. The constructed *sspA* null mutant was confirmed using Southern hybridization (data not shown).

**Fig 2:**
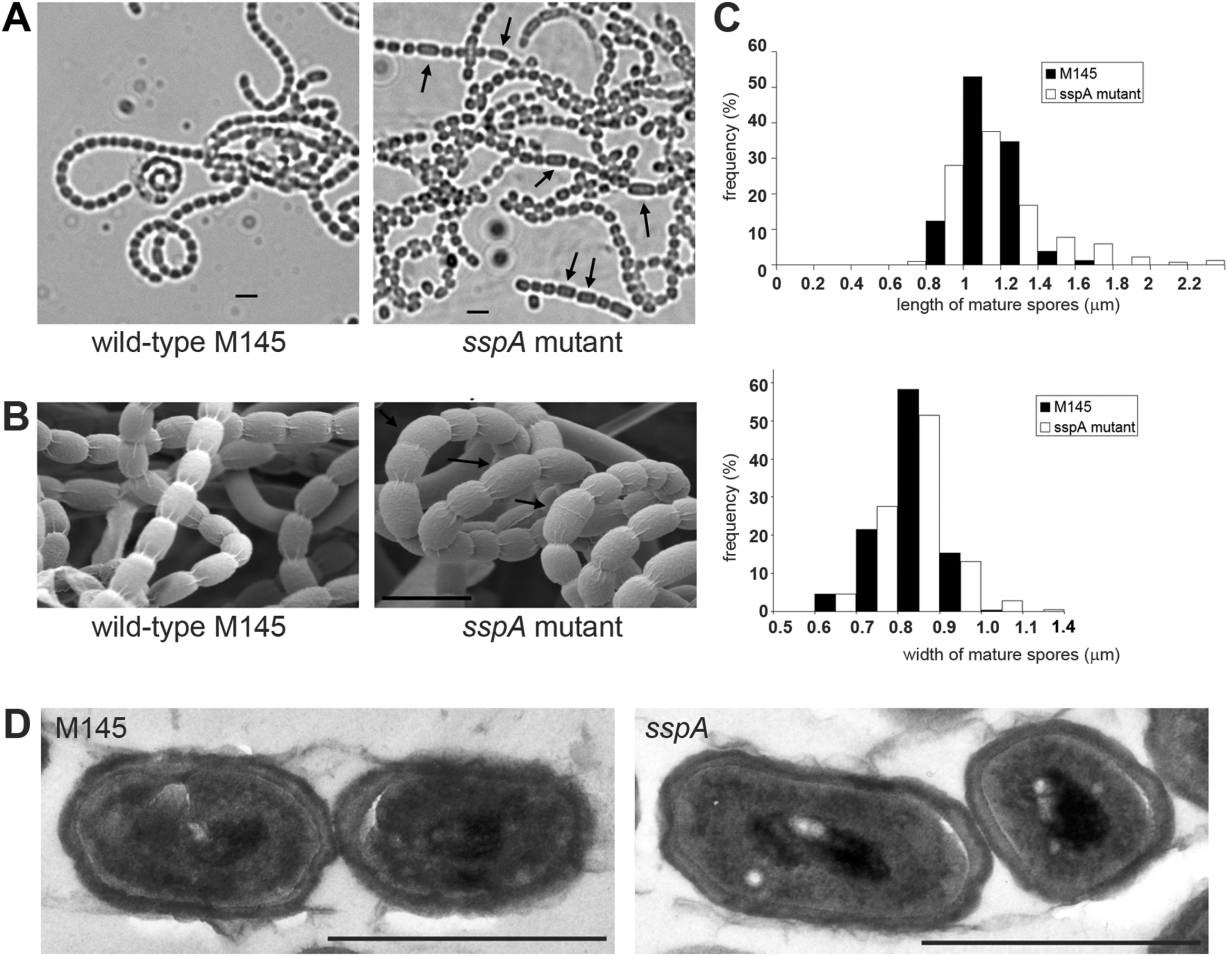
The *sspA* mutant generates irregularly sized spores. A. Wild-type M145 and the *sspA* mutant strains were grown in minimal medium for 72 h and the spores were viewed using phase-contrast microscopy. B. *S. coelicolor* wild-type M145 and the *sspA* mutant K55 were grown in SFM medium for 72 h and the spore morphology was assessed using scanning electron microscopy. Arrows mark spores longer than the wild-type average. The scale bar represents 2 μm. C. Histogram of the length and width of mature spores from *S. coelicolor* M145 (black) and the *sspA* mutant, K55 (white) grown in SFM medium for 72 h and viewed using phase-contrast microscopy. Measurements of ∼ 500 spores were analysed using the excel package. D. Transmission electron microscopy of the wild-type and *sspA* mutant spores, which were cultivated as in (B). Scale bars represent 1 μm.

The macroscopic phenotype of the *sspA* mutant was indistinguishable from the wild-type when grown both as confluent patches and as single colonies on a wide range of media including SFM medium, minimal medium with mannitol as carbon source or R5 medium. We also tested the sensitivity of the *sspA* mutant to osmotic, heat, cold or oxidative stress. No significant difference was detected when the wild-type or the *sspA* mutant was grown in the presence of 0.4 M KCl, 0.4 M NaCl or 0.8 M sucrose (osmotic stress), at 37°C (heat shock) or 16°C (cold shock) or using H_2_O_2_ disks. Testing the spores of the *sspA* mutant by treatment with 0.1% (v/v) Triton X-100 established that, unlike the *sigF* mutant (Potuckova *et al.*, 1995), spores of the *sspA* mutant were not sensitive to detergents (data not shown). However, microscopic observation identified subtle, but significant differences between spores of the *sspA* mutant and the wild-type. The length of 3- to 6-day-old mature spores of the *sspA* mutant was uneven, generating many spores longer than those of the wild-type, when grown in minimal medium supplemented with mannitol (Fig. 2). In addition, there were differences not only in spore size but also in spore shape between the two strains. While wild-type spores became ovoid following sporulation septation, spores of the *sspA* mutant were less rounded and rectangular-shaped, suggesting that spore maturation was affected in the *sspA* mutant (Fig. 2A). Interestingly, the length defects were detectable on several different media, including minimal medium and SFM medium. However, the ‘rectangular’ spore shape of the *sspA* mutant was most pronounced when the strain was grown in minimal medium (Fig. 2A), but less so in SFM medium (Fig. 2B). Statistical analysis of the length and width of mature spores from 3-day-old cultures grown in SFM medium confirmed that spore length of the *sspA* mutant varied much more than that of the wild-type (Fig. 2C). The mean length of mature spores was 1.17 μm for the wild-type and 1.18 μm for the *sspA* mutant with standard deviation (SD) of 0.14 and 0.31 respectively. The spore length varied between 0.69 μm and 2.47 μm for the *sspA* mutant, compared to the smaller range, between 0.87 μm and 1.75 μm, observed for the wild-type strain. The higher variance was specific to the length of the *sspA* spores as the spore-width of both strains was similar with a mean of 0.83 μm, SD = 0.08 (Fig. 2C). Consistent with these measurements, the wild-type spores typically exhibited ovoid shape in SFM medium. In contrast, the wider size range of spore-length of the *sspA* mutant resulted in a variety of shapes including spherical, ovoid and elongated rods (Fig. 2B and D). Interestingly, the *sigF* mutant produces smaller, spherical spores with thinner cell wall and less condensed chromosomes than that of the wild-type (Potuckova *et al.*, 1995). However, the spore wall and the condensed chromosomes of the *sspA* mutant were very similar to those of the wild-type, when assessed using transmission electron microscopy (Fig. 2D). This suggests that apart from the spherical spore shape, the characteristic *sigF* phenotype is not due to the lack of SspA.

**Fig 3:**
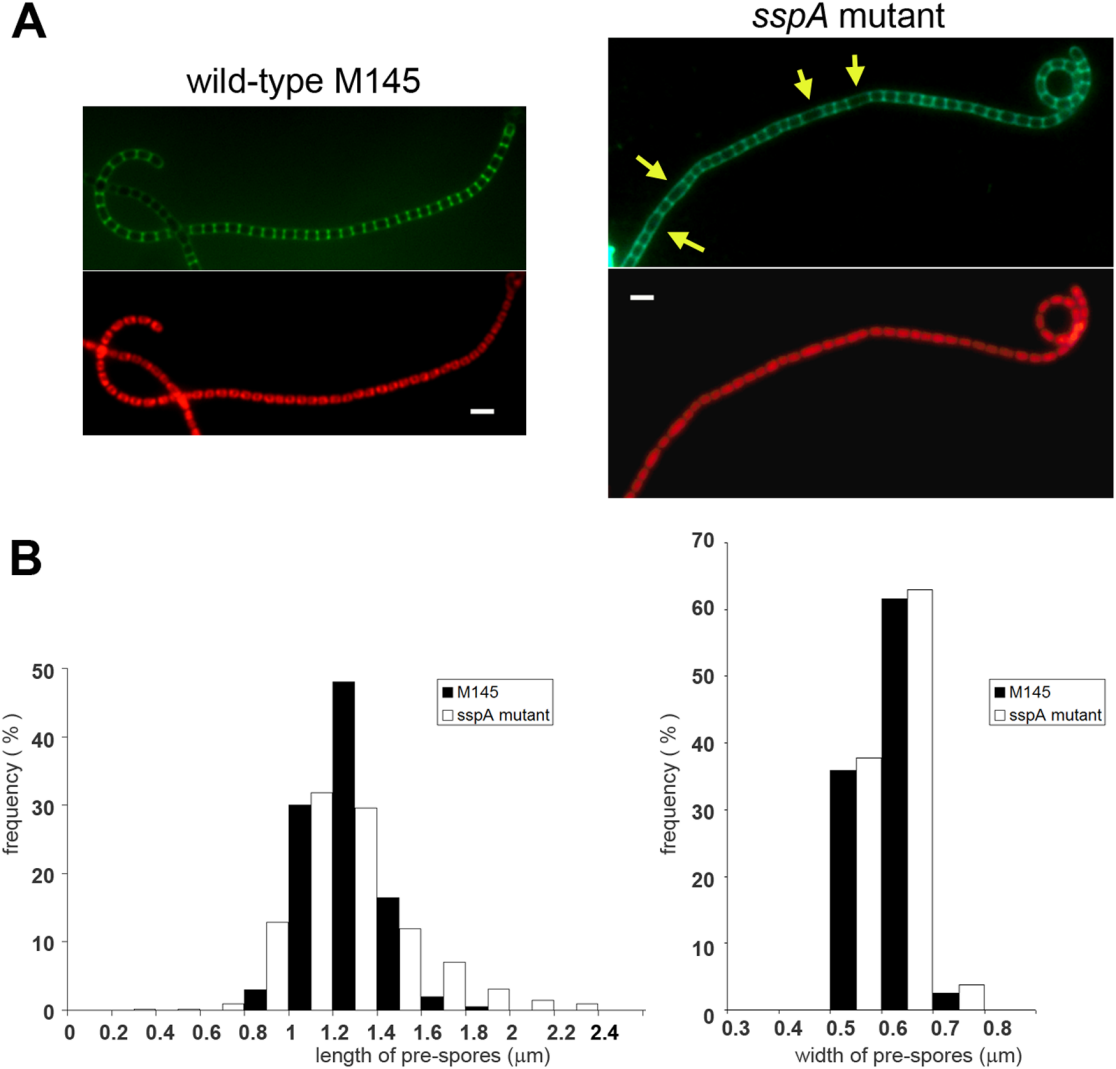
Septum formation is irregular in the *sspA* mutant. A. Early spore chains of the wild-type M145 and the *sspA* mutant, K55, were viewed using laser-scanning confocal microscopy after staining the cell wall with WGA-Alexa488 (green; top panel) and the chromosomes with propidium iodide (PI; red; bottom panel) after growth for 48 h in SFM medium alongside microscope coverslips to allow viewing of aerial development and sporulation. Yellow arrows mark aberrant septum formation in the *sspA* mutant. B. Histogram of the length and width of pre-spores within spore chains of the wild-type (black) and *sspA* mutant (white) strains. Samples were generated and viewed as in (A). Measurements of ∼ 550 spore compartments were analysed using the excel package.

To test whether the differences in spore length of the mature spores were the result of inaccurate septum placement or altered spore maturation, we tested spores at earlier stages, after 48 h growth, when sporulation septa were formed. Septa were stained with Alexa488 conjugate of Wheat Germ Agglutinin (WGA-Alexa488) and propidium iodide staining was used to visualize chromosomal DNA (Fig. 3). Measuring ∼ 500 pre-spore compartments for each strain, the mean length of pre-spores was slightly shorter, 1.26 μm, for the *sspA* mutant compared to 1.28 μm for the wild-type strain. While the mode was the same for both strains, the distance between septa of the *sspA* mutant had a much higher variance (0.08 versus 0.02) and higher standard deviation (0.29 versus 0.16). Pre-spore length varied over a wider range between 0.34 μm and 2.38 μm for the *sspA* mutant and between 0.81 μm and 1.99 μm for the wild-type strain, suggesting that lack of SspA affected both early septum placement and later spore maturation. There was no difference in the width of the hyphae at the time of septation (Fig. 3B) with a mean of 0.63 μm and SD = 0.05. The *sspA* mutant phenotype was fully complemented by the introduction of the *sspA* gene *in trans* using the single copy, integrative plasmid pMS82 (Gregory *et al.*, 2003), which confirms that the observed phenotype was due to lack of SspA.

### The *sspA* gene is transcribed from a single, *sigF*-dependent promoter

**Fig 4:**
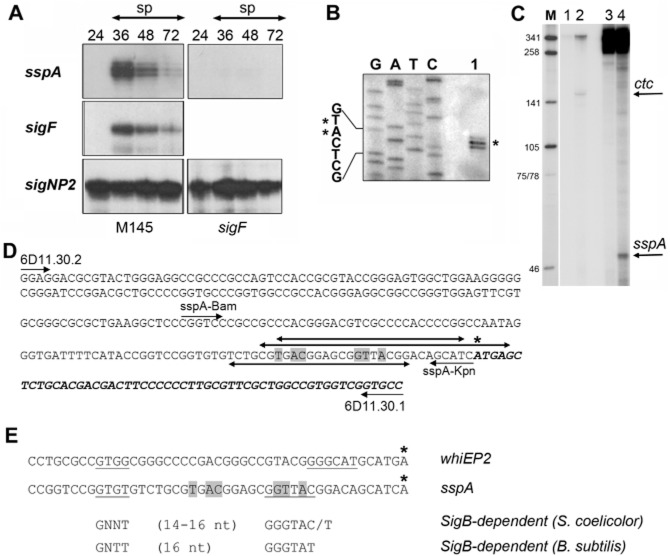
Transcription of *sspA* is *sigF* dependent. A. S1 nuclease analysis was performed using RNA samples collected at distinct developmental stages of *S. coelicolor* wild-type M145 (left) or *sigF* mutant (right) strains. Three different probes specific to *sspA*, *sigF* and *sigNP2* were used, this latter as a control to assess RNA integrity in the samples. sp indicated sporulating samples as was assessed by microscopy. B. Forty micrograms of RNA from 72-h-old wild-type (M145) samples, grown on SFM medium, were used together with a radioactively labelled probe containing the presumed transcriptional start for *sspA* together with a sequencing ladder to identify the exact transcriptional start point. C. *In vitro* run-off transcription was performed by RNA polymerase core enzymes (lanes 1 and 3) and RNA polymerase holoenzyme containing His-SigF (lanes 2 and 4) using the templates *ctc* (lanes 1 and 2) or *sspA* (lanes 3 and 4). The 155 nt *ctc*-specific transcript and the 54 nt long *sspA* transcripts are marked with arrows. The sizes of the marker DNA fragments generated using pUC19 digested with Sau3AI (M) are shown in basepairs. D. Sequence of the probe used for S1 nuclease analysis is shown. The starts of horizontal single arrows mark the 5′ ends of the oligonucleotides used. An asterisk marks the transcription initiation site that coincides with the translational start site. Translated sequences are italicized. The double headed arrows mark the regions protected by cell extracts (below the sequence; see also Fig. 6) or by purified BldD (above the sequence; see also Fig. 7) in DNase I footprinting assays. Grey highlighting indicates the most highly conserved nucleotides of the consensus BldD target sequence (den Hengst *et al.*, 2010). E. Putative promoter sequence of *sspA*. The promoter of *sspA* is compared to the SigF-dependent promoter, *whiEP2* together with the predicted consensus for SigB of *S. coelicolor* (Lee *et al.*, 2004) and the established consensus for SigB of *B. subtilis* (Petersohn *et al.*, 1999). Asterisks mark the transcription start points. The −10 and −35 promoter sequences are underlined and the BldD box (den Hengst *et al.*, 2010) is highlighted in grey.

SspA was identified from the 2D-PAGE analysis as a protein missing from the *sigF* mutant. To confirm that expression of *sspA* was indeed *sigF* dependent, we monitored *sspA* transcription using S1 mapping. mRNA was extracted from both wild-type, M145 and *sigF* mutant strains and samples were taken at various time points during morphogenesis including vegetative growth, aerial growth and sporulation. Using S1 mapping we were able to show that *sspA* was transcribed from a single, developmentally regulated promoter. The onset of *sspA* transcription coincides with that of *sigF* transcription. We also showed that *sspA* transcription was dependent upon *sigF* since *sspA* transcription was absent from the *sigF* mutant (Fig. 4).

The single transcription start point for *sspA* was determined using high-resolution S1 mapping (Fig. 4). Interestingly, the transcription start point for *sspA* is identical to its translational start point. Initiation of translation in bacteria involves binding of the 16S ribosome to the target mRNA, at a sequence of up to 6 ribonucleotides, which precedes the translational start codon. This sequence, called the Shine–Dalgarno sequence, base pairs with a region of the 16S RNA of the small ribosomal subunit. However, leaderless translational initiation, where both transcription and translation are initiated at the same position, is widely represented among bacteria and archaea (Zheng *et al.*, 2011). Leaderless mRNA has been documented for 13 *S. coelicolor* genes (Zheng *et al.*, 2011), including *absA* (Anderson *et al.*, 2001), *whiH* (Ryding *et al.*, 1998) or *vanH, vanK* and *vanR* (Hong *et al.*, 2004), just to name a few. It is not clear what the significance of the leaderless transcripts is in bacteria in general but the faithful translation of leaderless mRNAs in heterologous systems suggests that translation of leaderless mRNA is conserved among all kingdom of life (Moll *et al.*, 2002).

### SigF directs transcription from the *sspA* promoter of *S. coelicolor in vitro*

Beside *sspA,* the only other *sigF*-dependent promoter identified is *whiEP2*, which drives transcription of *SCO5321*, encoding an aromatic hydrolase involved in the biosynthesis of the spore-specific, grey pigment of *S. coelicolor* (Yu and Hopwood, 1995; Kelemen *et al.*, 1998). The *sigF-*dependent transcription from *whiEP2* was proposed to require a transcriptional activator, because RNA polymerase holoenzyme containing SigF alone failed to initiate transcription from *whiEP2, in vitro* (Kelemen *et al.*, 1998)*.* In order to test whether SigF was sufficient for *sspA* transcription, an *in vitro* transcription assay was performed. Previously we have used non-tagged SigF that was overexpressed and purified from *E. coli* (Kelemen *et al.*, 1998). Here, for easy purification, we overexpressed SigF with an N-terminal His extension in *E. coli* using the vector pET28a (Novagen). During the purification trials His-SigF was found in the insoluble fraction and was purified using denaturing conditions of the Ni-NTA kit (Qiagen). In *B. subtilis ctc* transcription is initiated by SigB (Igo and Losick, 1986) and the fact that *ctc* promoter activity does not require any additional transcription factors has made this promoter a commonly used template when testing the activities of SigB-like sigma factors, such as SigF (Kelemen *et al.*, 1998) or SigH (Viollier *et al.*, 2003) of *S. coelicolor*. In an *in vitro* run-off transcription assay, core RNA polymerase from *E. coli* together with His-SigF was sufficient to produce a specific transcript from a *ctc* template and also from an *sspA* template (Fig. 4C). Thus *sspA* is the first confirmed direct target for the RNA polymerase sigma factor SigF.

### SspA is expressed in the spores

**Fig 5:**
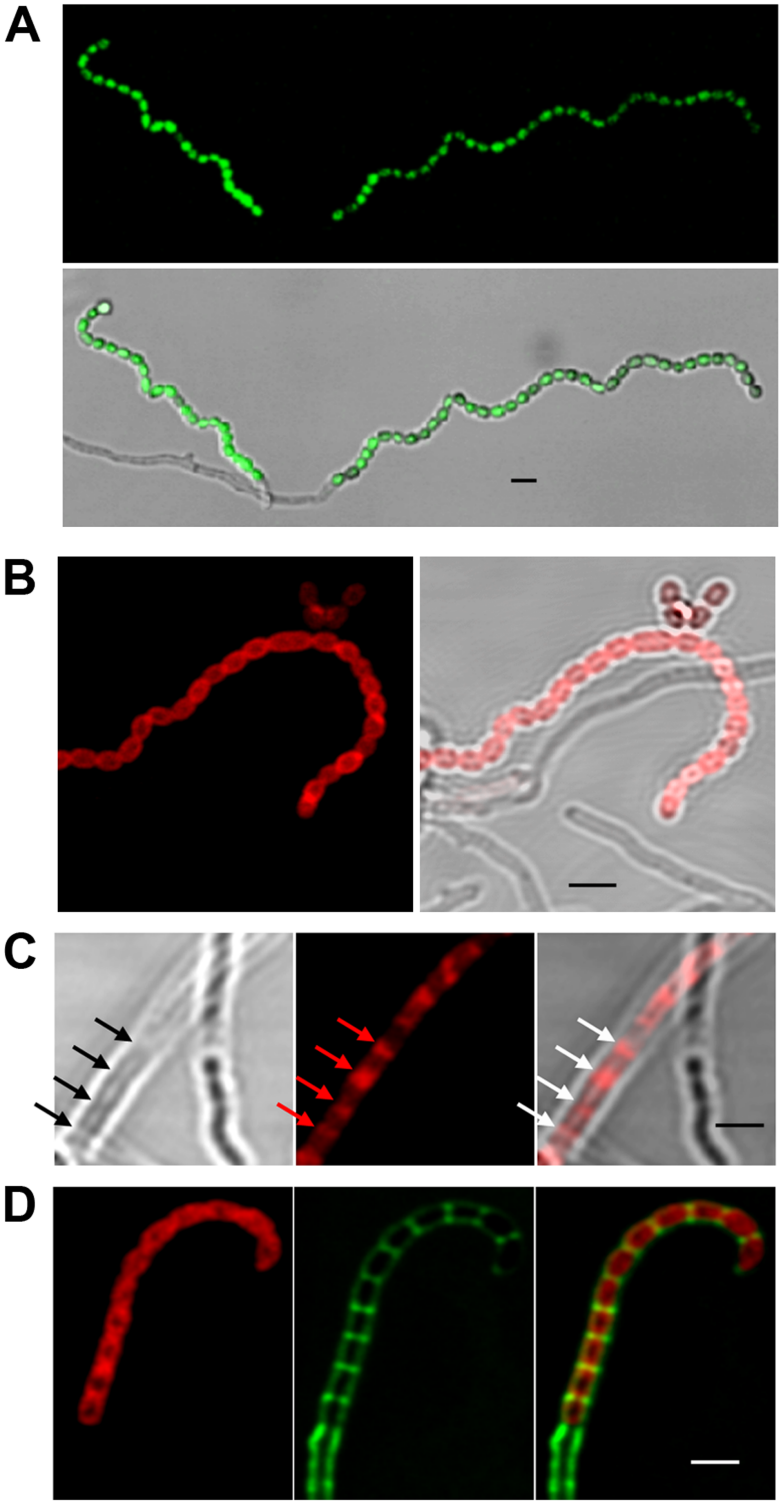
Monitoring SspA expression during sporulation. A. *S. coelicolor* M145/pK37, carrying *sspAP–egfp*, where Egfp is expressed from the *sspAP* promoter, was grown for ∼ 72 h in SFM medium alongside a coverslip and the samples were viewed by laser-scanning confocal microscopy. The green fluorescence image is above the overlaid image with the bright-field view. Scale bar represents 2 μm.B–D. *S. coelicolor* M145/pK39, expressing SspA–mCherry from pK39 integrated at the native chromosomal location of *sspA*, was grown for 72 h (B and D) or 44 h (C) in SFM medium and the samples were viewed by laser-scanning confocal microscopy. B. The red fluorescence image (left) is shown together with the overlaid image with the bright-field view (right). C. The bright-field view (left), the red fluorescence image (middle) and the overlaid image (right) are shown. Arrows mark the positions of developing septa. D. Samples were stained using WGA-Alexa488 (green, middle). The red fluorescence image (left) is shown together with the overlaid image (right). Scale bars represent 2 μm.

The S1 nuclease assays confirmed that *sspA* transcription temporally coincided with sporulation and SspA was purified from spore extract. To confirm the spatial location of *sspA* transcription and to monitor SspA production we generated both transcriptional and translational fluorescent protein fusions. The transcriptional fusion was generated using the plasmid pIJ8660 carrying the *egfp* gene downstream of a DNA fragment containing the *sspA* promoter, *sspAP,* but not the translated DNA region. This *sspAP–egfp* fusion was then introduced into *S. coelicolor* M145 and the green fluorescence was monitored throughout development. The *sspAP*-dependent fluorescence was detected in the spore chains, but there was no detectable fluorescence in the vegetative hyphae or the non-sporogenic part of the aerial hyphae, the subapical stem compartment (Fig. 5A).

**Fig 6:**
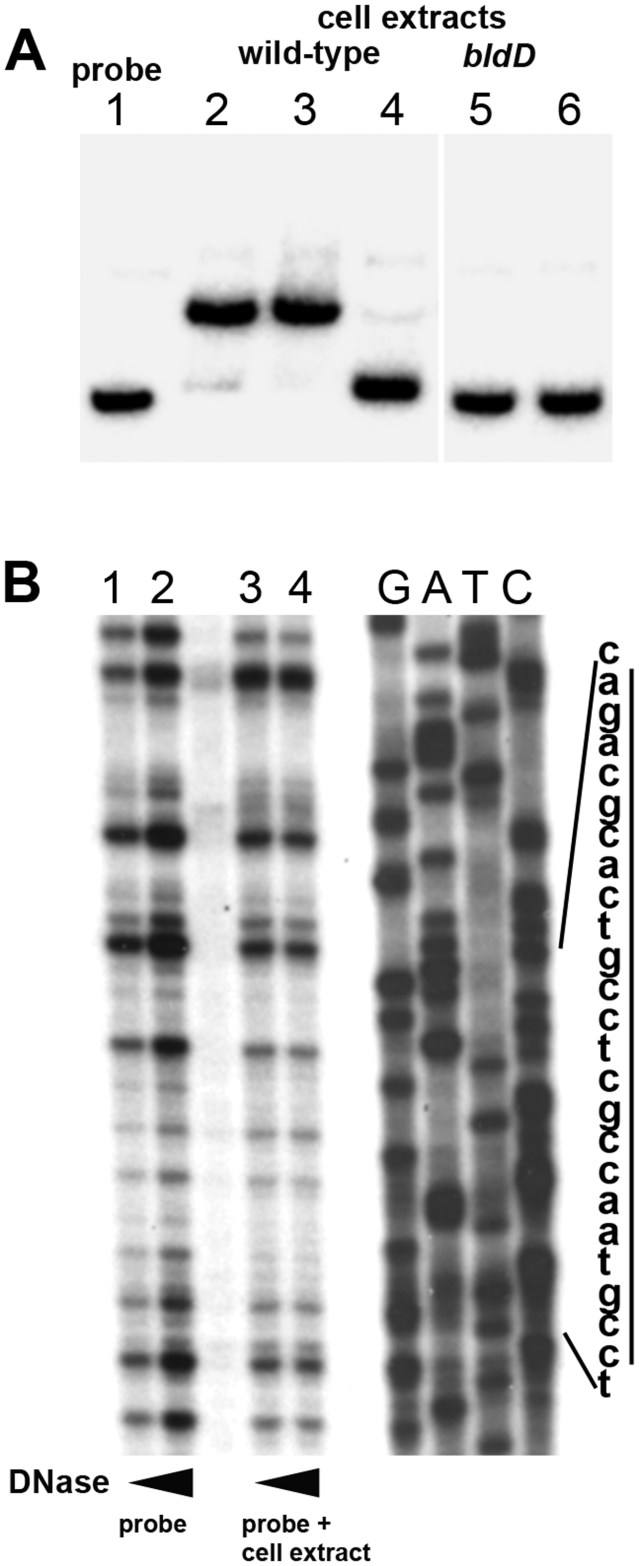
The *sspA* promoter is targeted by a putative repressor. A. Electrophoretic mobility shift assay. A radiolabelled DNA fragment containing the *sspA* promoter (lane 1) was incubated with increasing amounts (5 and 10 μg of protein) of cell extract from samples collected at an early developmental stage comprising of vegetative mycelium of wild-type *S. coelicolor* (lanes 2–3), or of the *bldD* mutant (lanes 5–6). The specificity of the protein–DNA interaction was confirmed by using 10 μg of protein extracts from the wild-type strain (as in lane 3) with the addition of a 50-fold excess of cold probe (lane 4). B. DNase I footprinting of the *sspA* promoter. The probe (as in A) was treated with increasing amount of DNase I either in the absence (lanes 1–2) or in the presence of 20 μg of protein extracts from wild-type *S. coelicolor* extracts (lanes 2–4). The generated fragments were analysed next to a sequencing ladder. The protected region is marked by a bar.

**Fig 7:**
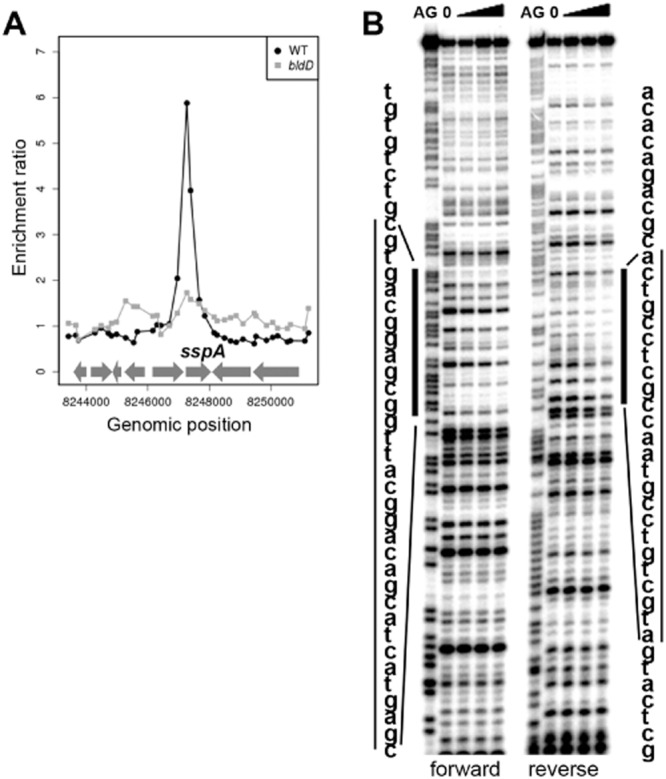
BldD targets the *sspA* promoter. A. BldD ChIP-chip data for the 8 kb region spanning the *sspA* locus in wild-type *S. coelicolor* (black circles) and the *bldD* mutant (grey squares). DNA obtained from immunoprecipitation of BldD was labelled with Cy3 and hybridized to DNA microarrays together with a total DNA control that was labelled with Cy5 (den Hengst *et al.*, 2010). Data generated by den Hengst were retrieved from the Gene Expression Omnibus (GSE23401) and were plotted as Cy3/Cy5 ratios (*y*-axis), as a function of chromosome location around *sspA* (*x*-axis). B. DNase I footprinting analysis of BldD binding to the promoter region of *sspA*. 5′ end-labelled probes were incubated in the presence of 0, 0.5, 1.0 or 2.0 μM BldD and subjected to DNase I footprinting analysis as described in den Hengst *et al.* (2010). Footprints are flanked by Maxam and Gilbert sequence ladders (AG). Protected regions are marked by bars.

To establish localization of the produced protein, we introduced an SspA–mCherry fusion into *S. coelicolor* M145 using pK39, a derivative of pIJ8668 (Sun *et al.*, 1999). Integration of pK39 into the chromosomal *sspA* site via homologous recombination replaced the native *sspA* gene with an *sspA–mCherry* allele in such a way that no wild-type SspA was produced and the fusion protein was expressed from the native *sspA* promoter. The spore morphology of *S. coelicolor* M145 carrying pK39 resembled that of the wild-type strain (data not shown), confirming that SspA–mCherry was functional. Monitoring fluorescence from the SspA–mCherry translational fusion confirmed that SspA localized to the spore walls (Fig. 5B). Hence the fluorescence SspA–mCherry signal is ring-like (Fig. 5B) in contrast to the fluorescence from the *sspAP–egfp* fusion that fills the cytoplasm (Fig. 5A). More interestingly, we could detect SspA–mCherry at an earlier stage, at the developing sporulation septa, which was apparent by the regularly spaced invagination of the hyphal wall (Fig. 5C). To colocalize SspA–mCherry and the developing septa we used WGA-Alexa488 staining (Fig. 4D). Interestingly, at the time when fluorescent WGA-Alexa488 was detectable in sporulation septa, SspA–mCherry was already found around the spores (Fig. 5D). This suggests that the early, septal SspA–mCherry localization precedes the stage of septation that is detectable by wheat germ agglutinin.

### The promoter of *sspA* is targeted by BldD

To test for any potential transcriptional regulators targeting the *sspA* promoter, we performed electrophoretic mobility shift assays. Cell lysate prepared from the wild-type strain grown for 28 h on SFM medium shifted a radiolabelled DNA fragment that contained the *sspA* promoter (Fig. 6A), suggesting the presence of a regulator of the *sspA* promoter. The interaction between the *sspA* DNA fragment and the putative regulator appeared specific, since the gel shift was detected in the presence of non-specific DNA [poly(dI-dC)]. Moreover, excess cold probe DNA abolished the shift (Fig. 6A). Since the DNA-binding activity was detected in cell extracts made from samples of vegetative mycelium, where *sspA* is not expressed, it seemed likely that this putative regulator might function as a repressor of *sspA* transcription.

To determine the binding site of this putative regulator, we carried out DNase I footprinting, using the same *sspA* DNA fragment used for the S1 mapping and EMSA experiments. The crude protein extract from vegetative mycelium of the wild-type strain weakly protected a region of the *sspA* promoter (Fig. 6B). A sequence in the protected region resembled the ‘BldD box’ [TnACnnnnnGTnA], the palindromic consensus sequence targeted by the BldD repressor protein (den Hengst *et al.*, 2010), raising the possibility that the *sspA* promoter was a direct target of BldD. Further, an equivalent cell extract from a *bldD* null mutant failed to shift the *sspA* probe in a gel-shift assay (Fig. 6A). Chromatin immunoprecipitation microarray (ChIP-chip) analysis showed that *sspA* is a direct target of BldD *in vivo* (Fig. 7A) (den Hengst *et al.*, 2010). To confirm and extend this analysis, the BldD binding site in the *sspA* promoter was mapped precisely using DNase I footprinting (Fig. 7B). Incubation with purified histidine-tagged BldD protected a ∼ 30 bp region including the sequences that were protected by the cell extracts from vegetative mycelium of the wild-type strain (Fig. 4D). The overlapping protected region spans the putative BldD box and the −10 promoter site, a location consistent with BldD functioning as a repressor of the *sspA* promoter (Fig. 4D).

## Discussion

### SspA controls spore development by affecting septum placement

The SspA protein has been identified as an abundant protein in wild-type spores and is absent from the spores of a *sigF* null mutant. Bioinformatic analysis identified both an N-terminal signal sequence and a lipoprotein signature suggesting that SspA is exported through the cell membrane and attached to it, presumably from the outside. The rest of the SspA protein sequence comprises of two PepSY domains that are widespread in bacteria and fungi and found often in the propeptide domains of M4 proteases (Yeats *et al.*, 2004). Where examined, the PepSY motif of the propeptide was shown to function as an intramolecular inhibitor preventing premature activation of the protease (Braun *et al.*, 2000; Tang *et al.*, 2003; Gao *et al.*, 2010). While the M4 peptidases are well characterized, no clear biological role was established for those PepSY-family proteins that do not possess additional domains with enzymatic activity. The presence of the PepSY domain in this diverse family of secreted and cell wall-associated proteins suggested a regulatory role for protease activity in the local environment of the cell (Yeats *et al.*, 2004). However, recent studies of PepSY-domain transmembrane proteins suggested that these domains can regulate the activity of proteins other than proteases. For example, the PepSY proteins BqsP and BqsQ of *Pseudomonas aeruginosa* are proposed to regulate the activity of a two-component system that senses extracellular Fe(II) (Kreamer *et al.*, 2012). Similarly, the PepSY protein YycI regulates the YycFG two-component system in *B. subtilis* (Szurmant *et al.*, 2007). Structural analysis proposed a common structural fold for the PepSY domain, the beta-lactamase inhibitor protein fold (BLIP) and two other protein folds, suggesting that all these protein folds function as inhibitors by binding a partner domain that is located either within the same protein or on a separate protein (Das *et al.*, 2010).

Interestingly, one of the cortex-lytic enzymes in *B. subtilis*, SleB, is associated with YpeB, a protein with two copies of the PepSY domain (Boland *et al.*, 2000). YpeB has been implicated in the control of a peptidoglycan amidase, SleB, important during spore germination (Atrih and Foster, 2001). YpeB was required for SleB localization (Chirakkal *et al.*, 2002) and it was suggested that YpeB might recruit SleB to the spore cortex. However, recently the inhibition of SleB by YpeB was demonstrated, suggesting that the PepSY protein YpeB controls SleB activity (Li *et al.*, 2013) perhaps by inhibiting the premature activation of the SleB lytic activity in the spores of *B. subtilis.*

SspA possesses two PepSY domains but lacks any catalytic peptidase domain, so its domain organization is very similar to that of YpeB. However, germination of the *sspA* mutant spores was not affected (data not shown). Instead, lack of SspA altered septum formation on all media tested and spore maturation when grown on minimal medium. Therefore, we hypothesize that SspA might control the activity of a specific peptidase or peptidoglycan hydrolase involved in either septum formation or spore maturation. Septum positioning depends on the key cell division protein FtsZ that assembles into 50–100 regularly spaced FtsZ rings marking future septum sites (Schwedock *et al.*, 1997). In *E. coli* or *B. subtilis*, FtsZ positioning is regulated by FtsZ antagonist proteins. These include the MinCD complex located at cell poles promoting FtsZ ring formation at mid-cell and the chromosome associated SmlA or Noc proteins, which prevent FtsZ ring formation and therefore septum formation over the chromosomes (Adams and Errington, 2009; Lutkenhaus, 2012). In *Streptomyces* no apparent homologues of either the Min system or nucleoid occlusion proteins have thus far been identified. Instead, formation of the FtsZ rings is under a positive control by SsgB in *Streptomyces* (Willemse *et al.*, 2011; Jakimowicz and van Wezel, 2012) while FtsZ protein levels are negatively affected by CrgA (Del Sol *et al.*, 2006, Del Sol *et al.*, 2003). Following on from the formation of the FtsZ rings, very little is known about the recruitment of specific enzymes for septum synthesis in *Streptomyces.* Interestingly, the FtsW–FtsI protein pair, which is established in septum formation in *E. coli*, was proposed for FtsZ ring stabilization in *Streptomyces* (Mistry *et al.*, 2008; Bennett *et al.*, 2009; McCormick, 2009). In addition, the SsgA-like proteins (SALPs) have been implicated in both septum positioning (SsgA and B) and in septum formation (SsgC-G) (Noens *et al.*, 2005; Willemse *et al.*, 2011; Jakimowicz and van Wezel, 2012).

The late sigma factor, SigF is associated with the control of spore maturation, which is a post-septation event. However, the *sigF* null mutant produces smaller spores than the wild-type (Potuckova *et al.*, 1995). This could be the result of incorrect septum positioning and possibly altered FtsZ placement suggesting that SigF is active when septa are formed. Similarly, the uneven septum positioning in the *sspA* mutant could arise from altered FtsZ positioning. Alternatively, SspA might influence the recruitment of cell wall lytic or synthetic enzymes after FtsZ ring formation. Interestingly, monitoring SspA localization using an SspA–mCherry fusion confirmed that in addition to its presence in mature spores, SspA accumulated at sporulation septa (Fig. 5B). This suggests that SspA functions as early as septum formation. It will be important to establish the potential link between SspA and the Fts proteins (FtsZ, FtsW and FtsI), CrgA and the SALPs.

After the completion of septation, spore wall synthesis is governed by the cytoskeletal proteins MreB and Mbl (Mazza *et al.*, 2006; Heichlinger *et al.*, 2011). MreB polymers first assemble at sporulation septa, followed by spreading to the entire spore wall (Mazza *et al.*, 2006). This pattern is reminiscent of SspA localization, raising the possibility that SspA positioning might be MreB-dependent. On the other hand, lack of SspA affects septation while MreB only controls post-septational events (Mazza *et al.*, 2006) suggesting that SspA might function *prior* to MreB assembly during spore development. A recent search for MreB partner proteins established a complex interaction pattern among members of the proposed ‘*Streptomyces* spore wall synthesizing complex’, SSSC (Kleinschnitz *et al.*, 2011). A knockout mutant of SCO2097, a putative membrane protein identified among the SSSC proteins, produced elongated spores with sensitivity to heat and cell wall-damaging agents (Kleinschnitz *et al.*, 2011). Interestingly, these elongated spores resemble those of the *sspA* mutant; however, the latter did not exhibit sensitivity to lysozyme or heat (data not shown). In addition, spore wall hydrolytic enzymes have also been shown to affect spore shape (Haiser *et al.*, 2009). It will be of interest to test whether any of the penicillin-binding proteins of the SSSC, including SCO3901, SCO3580 and FtsI (Kleinschnitz *et al.*, 2011) or any of the hydrolytic enzymes, RpfA, SwlA, SwlB and SwlC (Haiser *et al.*, 2009) are targets of or partnered by SspA.

### Transcription of *sspA* is under the control of the sigma factor, SigF and the principal developmental regulator, BldD

Both the production of SspA protein and transcription of *sspA* are dependent on *sigF in vivo,* in *S. coelicolor* (Figs 1 and 4). Moreover, *in vitro* transcription assays (Fig. 4) confirmed that SigF is sufficient to initiate transcription from the *sspA* promoter. Hence, in this report we have presented the first example of a SigF target promoter, *sspAP* where transcription is initiated by RNA polymerase holoenzyme containing the sigma factor, SigF in the absence of any activator. SigF belongs to a group of nine RNA polymerase sigma factors (SigB, F, G, H, I, K, L, M and N) that control response to environmental stresses (SigB, H, I, L and M) or morphological differentiation (SigH, F and N) or in some cases both (SigH and SigB) in *S. coelicolor* (Potuckova *et al.*, 1995; Cho *et al.*, 2001; Kelemen *et al.*, 2001; Sevcikova *et al.*, 2001; Viollier *et al.*, 2003; Lee *et al.*, 2005). Members of this, so called, SigB-family of *S. coelicolor* resemble the general stress response sigma factor SigB of *B. subtilis* (see review, Price, 2000). Predictably, the putative promoter sequence of *sspA* (GTGT-16N-GGTTAC) resembles the consensus target sequence of SigB both in *S. coelicolor* and in *B. subtilis* (Fig. 4E; Petersohn *et al.*, 1999; Price, 2000; Lee *et al.*, 2004). Interestingly, the weak similarity between the two *sigF*-dependent promoters, *sspA* and *whiEP2,* together with the unusually long spacer between the −10 and −35 sequences of *whiEP2* might explain why SigF was not sufficient to initiate transcription from *whiEP2 in vitro* (Kelemen *et al.*, 1998). Identification of further SigF target promoters is paramount in order to establish a consensus SigF target sequence and to address the fundamental question of how members of the SigB-family with potentially overlapping promoter specificity can control distinct sets of genes *in vivo*. One such mechanism could include specific activators, as it was proposed for the promoters *whiEP2* and *nepA,* which are putative targets for SigF and SigN respectively (Kelemen *et al.*, 1998; Dalton *et al.*, 2007).

Alternatively, transcriptional repressors could restrict expression of target genes both in time and in space, allowing limited access to promoter sites by cognate sigma factors. Interestingly, gel shift assays together with DNase I footprinting demonstrated a DNA binding activity from wild-type cell extracts collected at early stages of development, when *sspA* was not expressed, suggesting a putative repressor targeting *sspA* transcription. Both *in vivo* and *in vitro* experiments confirmed that this repressor is BldD, a key developmental regulator of *Streptomyces* morphogenesis. *bldD* was among the first developmental genes identified in the study of morphological differentiation in *Streptomyces* (Merrick, 1976). The *bldD* mutant fails to progress to aerial development and is also blocked in the production of several secondary metabolites (Elliot *et al.*, 1998). BldD, a small DNA-binding protein, has been shown to target, and mainly repress, the transcription of developmental genes, such as the sigma factor genes, *whiG, bldN* and *sigH,* during early development while co-ordinating the timing and, in some cases, the location of their expression at later stages of differentiation (Elliot *et al.*, 2001; Kelemen *et al.*, 2001). Recently, the genome-wide BldD regulon has been extensively defined by chromatin immunoprecipitation-microarray analysis identifying ∼ 167 transcription units targeted by BldD (den Hengst *et al.*, 2010). The location of the BldD binding site at the *sspA* promoter is consistent with BldD functioning as a repressor of *sspA,* perhaps by blocking access of other SigB-like sigma factors to the *sspA* promoter during early developmental stages. Surprisingly, microarray analysis showed that *sspA* transcription was not upregulated but absent in the *bldD* null mutant (den Hengst *et al.*, 2010). This suggests that either the *sspA* promoter is recognized exclusively by SigF, which is naturally absent from the *bldD* mutant, or, more likely, no other SigB-like sigma factors capable of initiating *sspA* transcription were active under the conditions of the microarray analysis. Interestingly, one of the BldD targets identified in this analysis is the gene encoding SCO4677 (den Hengst *et al.*, 2010), which has been demonstrated to bind SigF, potentially functioning as an anti-sigma factor (Kim *et al.*, 2008). Thus BldD is linked to the *sigF* regulon via *sspA*, and perhaps also via SCO4677. Moreover, BldD has also been shown to target cell division genes (den Hengst *et al.*, 2010), such as *ftsZ*, *ssgA* and *ssgB* or the *smeA-ssfA* operon encoding a DNA translocase required for correct chromosome segregation during sporulation (Ausmees *et al.*, 2007). Hence, BldD appears to co-ordinate the expression of proteins that are required at the time of septum formation, including the expression of SspA.

## Experimental procedures

### Bacterial strains and growth conditions

*Escherichia coli* DH5α (Hanahan, 1983) was used for routine cloning. *E. coli* BW25113 carrying pIJ790 (Datsenko and Wanner, 2000) was the host for recombination between the extended apramycin resistance cassette and the target gene to generate knockout mutants. *E. coli* ET12567 (MacNeil *et al.*, 1992) containing pUZ8002 (Kieser *et al.*, 2000) aided the transfer of plasmids or cosmids from *E. coli* into *S. coelicolor* by conjugation. The strains of *S. coelicolor* used in this work are listed in Table 1. *Streptomyces* strains were grown at 30°C on SFM (soya flour medium containing 1% mannitol), MM (minimal medium) supplemented with mannitol (0.5%) solid media. pIJ82 is a hygromycin-resistant derivative of pSET152 (Bierman *et al.*, 1992) in which the apramycin resistance gene is replaced with the hygromycin resistance gene (Dalton *et al.*, 2007). All plasmids and oligonucleotides used in this work are shown in Tables 2 and 3 respectively.

**Table 1:** Bacterial strains used in this work

Strain	Genotype or description	Reference or source
*S. coelicolor*		
M145	SCP1^−^ SCP2^−^	Kieser *et al.* (2000)
K55	Δ*sspA*::*apr* derivative of M145	This study
J1984	Δ*sigF*::*thio* derivative of M145	Kelemen *et al.* (1998)
Δ*bldD*	Δ*bldD::apr* derivative of M600	Elliot *et al.* (2003b)
*E. coli*		
DH5α	Cloning	Invitrogen
ET12567/pUZ8002	Transferring plasmids or cosmids from *E. coli* to *S. coelicolor* by conjugation	MacNeil *et al.* (1992), Kieser *et al.* (2000)
BL21 (DE3)pLysS	Protein overproduction	Novagen
BW25113/pIJ790	Knockout generation	Datsenko and Wanner (2000)

**Table 2:** Plasmids used in this work

Plasmid	Genotype or description	Reference or source
pET28a	Vector for protein overproduction	Novagen
pIJ8660	This plasmid integrates as a single copy at the ΦC31 *attB* attachment site on the chromosome of *S. coelicolor*	Sun *et al.* (1999)
pIJ8668	This plasmid cannot replicate autonomously in *S. coelicolor* but it can integrate into the *S. coelicolor* chromosome via homologous recombination through the appropriate inserted sequences.	Sun *et al.* (1999)
pIJ82	This plasmid integrates as a single copy at the at the ΦC31 *attB* attachment site on the chromosome of *S. coelicolor*	Dalton *et al.* (2007)
pAT1	pIJ82 carrying the 1.3 kb *sspA* fragment	This study
pAT6	pUC18 carrying a 7.7 kb BamHI fragment of *sspA*	This study
pK37	pIJ8660 carrying the *sspAp–egfp* transcriptional fusion	This study
pK39	pIJ8668 derivative for the expression of the SspA–mCherry translational fusion when integrated into the *S. coelicolor* chromosome	This study
pET28-SigF	pET28a derivative for the overexpression of His-SigF from *E. coli*	This study

**Table 3:** Oligonucleotide primers used in this work

sspA-Bam	CTGAAGGATCCCGGTCCCGCCGCCCACGGGACG
sspA-Bam2	CTGAAGGATCCCAGCATCATGAGCTCTGCACGACG
sspA-Nde	GATACCATATGGTCCTTGTCGACCTTCTGGCCC
sspA-Kpn	GCAGAGGTACCGATGCTGTCCGTAACCGCTCCG
6D11.30.1	GGCACCGACCACGGCCAGCG
6D11.30.2	GGAGGACGCGTACTGGGAGG
6D11.301	GTGTGTCTGCGTGACGGAGCGGTTACGGACAGCATCATGATTCCGGGGATCCGTCGACC
6D11.302	GGCCCGCTGCTCCCGTCGCTGTCCGGAGGGGGTGACTCATGTAGGCTGGAGCTGCTTC
SIGN4	CTGGTGCGCCACCTGCTCGTCC
SIGN7	GGACATGCCACCCCCTTTGG
SIGF15	GAACATCCGGCGCACCAGGCG
SIGF16	CGGCGCGGGCGGTGCTTGAGGCGC
DIR	CGCCAGGGTTTTCCCAGTCACGACG
EGFPLINKER1	GATCCTCTAGACATATGGGCGGCGGCGGCGG
EGFPLINKER2	TACCGCCGCCGCCGCCCATATGTCTAGAG
7434_F1	CTGAAGGCTCCCGGTCCCGC
7434_R1	GTGCAGGCCGTCAGCAGCAG

### Preparation of protein extracts from spores

Spores were harvested from cultures grown on SFM medium according to Kieser *et al.* (2000) and were washed with 40 mM Tris pH 9.0; 1 mM EDTA; 1 mM EGTA buffer. Washed spores were transferred to a pestle and mortar submerged in liquid nitrogen (typically a 0.5 ml volume of spore pellet was used), and disrupted by thoroughly grinding in the presence of an equal volume of fine glass beads (106 microns; Sigma G8893). The frozen, ground spore powder was then transferred on ice into the above buffer containing 100 mM DTT and 4 mM Pefabloc SC protease inhibitor and protector solution (Roche). On warming to approximately 4°C, SDS was added to a final concentration of 2%, and proteins extracted by boiling for 10 min. Cell debris and glass beads were removed by centrifugation, and the protein extract was precipitated using the Amersham 2D Clean-Up kit (80-6484-51) according to the manufacturer's instructions. This step was vital for removal of SDS which interferes with subsequent separation by isoelectric focusing. The precipitated protein pellet was finally redissolved in denaturing UTCHAPS isoelectric focusing buffer [7 M urea, 2 M thiourea, 4% w/v CHAPS, 40 mM Tris, pH 9.0, 1 mM EDTA, 50 mM DTT, 4 mM Pefabloc SC protease inhibitor (Roche)]. Extracts were stored at −80°C until use.

### 2D gel-electrophoresis and protein identification

Protein extracts were separated by 2D gel-electrophoresis as previously described (Hesketh *et al.*, 2002). Briefly, proteins were separated in the first dimension for 120 000 volt-hours using 18 cm IPG strips pH 4.5–5.5 (Amersham Biosciences) using a Phaser isoelectric focusing unit (Genomic Solutions). Focused strips were separated in the second dimension using in-house fabricated 12.5% SDS-PAGE gels and the Investigator 5000 vertical format system from Genomic Solutions. Protein spots of interest were excised manually from colloidal Coomassie-stained gels, and identified by tryptic digestion and MALDI-TOF mass spectrometry as previously described (Hesketh *et al.*, 2002).

### Generation of the *sspA* knockout mutant

The *sspA* knockout mutant was generated using PCR targeting (Gust *et al.*, 2003) by replacing the entire *sspA* gene, excluding the translational start and stop codon, with the apramycin resistance cassette. The apramycin resistance gene and *oriT* was PCR-amplified using the primers 6D11.301 and 6D11.302 and was introduced into cosmid St6D11 in BW25113 (Datsenko and Wanner, 2000) carrying pIJ790 to replace *sspA* with the apramycin resistance cassette. The cosmid carrying the mutant allele was passaged through the *dcm-dam*-ET12567 strain (MacNeil *et al.*, 1992) containing pUZ8002 and was introduced into *S. coelicolor* M145 by conjugation. The double cross-over exconjugants were screened for resistance to apramycin and sensitivity to kanamycin. One of the *sspA* mutants, which was confirmed by Southern blot hybridization was designated K55.

### Complementation of the *sspA* mutation

A ∼ 7.7 kb BamHI DNA fragment containing the entire *sspA* gene was first moved from cosmid 6D11 into pUC18 and from this subclone, pAT77, a ∼ 1.3 kb BamHI–MluI fragment carrying exclusively the *sspA* gene was introduced, via several steps, into pIJ2925 (Janssen and Bibb, 1993) to generate pAT0. The *sspA* fragment was liberated from pAT0 using BglII, and introduced into the BamHI site of pIJ82 (Dalton *et al.*, 2007), to create pAT1. pAT1 was introduced into *S. coelicolor* M145 and K55 by conjugation and hygromycin-resistant exconjugants, carrying pAT1 integrated to the ΦC31 attachment site on the chromosome, were examined for their phenotype.

### S1 nuclease mapping

Mycelium grown on solid medium covered with cellophane discs was collected, and initial extracts were generated by grinding in liquid nitrogen. From these extracts RNA was prepared and S1 nuclease protection assays were performed according to Kieser *et al.* (2000) using 40 μg RNA in each assay. Probes labelled with ^32^P at their single 5′ end were generated using PCR with two oligonucleotides, one labelled at its 5′ end with [γ-^32^P]-ATP using T4 polynucleotide kinase. Probes were generated using the following oligonucleotide pairs: *sspA* probes using 6D11.30.1* and 6D11.30.2; *sigF* probes using SIGF15 and SIGF16*; *sigN* probes using SIGN4 and SIGN7* (asterisks mark the labelled oligonucleotides). DNA fragments protected by RNA were separated on 6% sequencing gels. The transcriptional start point of *sspA* was identified against a dideoxy-sequencing ladder (Amersham Pharmacia biotech; T7 sequencing™ kit) produced using the same labelled oligonucleotide used to generate the probes for S1 analysis.

### Generation of transcriptional and translational fusions

Two appropriate primers, sspA-Bam and sspA-Kpn, were used to generate a PCR product carrying a 100 bp sequence upstream of the *sspA* transcriptional start. This PCR product was introduced into pIJ8660 (Sun *et al.*, 1999) as a BamHI–KpnI fragment generating pK37, which was introduced into *S. coelicolor* M145 by conjugation. One of the representative apramycin-resistant exconjugants was used to monitor *sspA* transcription. *S. coelicolor* M145 carrying pK37 was propagated on medium containing apramycin to maintain selection for the presence of plasmid integrated in a single copy into the ΦC31 *attB* site in the *S. coelicolor* chromosome.

To monitor the localization of SspA–mCherry we constructed pK39. First a flexible linker encoding Gly_4_MetAla was introduced upstream of the *egfp* in pIJ8668 (Sun *et al.*, 1999) by cloning the annealed Egfplinker1 and Egfplinker2 primers into the BamHI–NdeI sites of pIJ8668. Then the *egfp* fragment was replaced by an *mCherry* fragment using NdeI and BsrGI restriction sites. The *sspA* coding sequences were generated by PCR using the primers sspA-Bam2 and sspA-Nde, which create BamHI and NdeI sites at the respective ends. The BamHI–NdeI *sspA* fragment was introduced to the linkered pIJ8668 derivative. This generated a translational *sspA–mCherry* fusion, which, when introduced to wild-type *S. coelicolor* M145, integrates via homologous recombination at the *sspA* site in the chromosome.

Fluorescent protein fusions were monitored using a Leica TCS SP2 laser-scanning confocal microscope with a 63×, 1.4NA oil-immersion objective.

### *In vitro* run-off transcription using His-SigF

In order to produce His-SigF the *sigF* gene was introduced into pET28a (Novagen) as an NdeI–EcoRI fragment from pIJ5894 (Kelemen *et al.*, 1998) that was generated previously to overexpress non-tagged SigF. The resulting clone, pK34, was moved into *E. coli* BL21 (DE3)pLysS (Novagen). Cultures of BL21(DE3)pLysS/pK34 were grown at 37°C to an OD_600_ 0.4 and, after induction with 1 mM IPTG, for a further 4 h. After harvesting the mycelium, His-SigF was purified under denaturing conditions (8 M urea) using the Ni-NTA Spin Kit (Qiagen) according to the manufacturer's instructions. Fractions eluted from the Ni-NTA column with a buffer of low pH (4.5) were dialysed against 50 mM NaH_2_PO_4_ and 50 mM NaCl, pH 8.0 buffer at 4°C. The His-SigF protein was analysed on an 8% SDS-PAGE and stored at −20°C in the presence of 20% glycerol.

*In vitro* run-off transcription was performed as described by Buttner *et al.* (1987) using 0.5 μg His-SigF, core RNA polymerase from *E. coli* (Cambio), [α-^32^P]-CTP (3000 Ci mmol^−1^; Amersham Biosciences) and an appropriate DNA template. The 340 bp EcoRI–BamHI fragment of pMI340 (Igo and Losick, 1986) was used to test transcription from the *B. subtilis ctc* promoter and was expected to produce a 155-nucleotide (nt) *ctc* transcript. The template carrying the *sspA* promoter was generated by PCR using the 6D11.30.1, DIR oligonucleotide pair and pAT6 as template. pAT6 carries a 7.7 kb BamHI fragment from cosmid St6D11 in the vector pUC18 and the DIR oligonucleotide anneals to pUC18 sequences. The 312 bp PCR product carried 229 bp *Streptomyces* sequence that was expected to generate an 54 nt *sspA* transcript *in vitro*.

### Electrophoretic mobility shift assays and DNase I footprinting

The labelled *sspA* probe from the S1 nuclease assays were used together with either cell extracts from *S. coelicolor* strains or BldD protein overexpressed and purified from *E. coli* (den Hengst *et al.*, 2010). *S. coelicolor* strains were grown on the surface of cellophane discs positioned onto SFM medium for 28 h producing a lawn of vegetative mycelium. The collected cells were lysed by sonication and the cell debris was removed by centrifugation at 15000 *g* for 20 min. Samples of the supernatant were incubated with the *sspA* probe at 30°C for 20 min in the presence of 1 μg Poly(dI-dC)●Poly(dI-dC) (SIGMA) in 25 mM HEPES pH 7.5, 4 mM DTT, 1 mM ATP, 10 mM Mg-acetate, 4% glycerol buffer and the protein:DNA complexes were analysed on a 4% acrylamide gel, followed by autoradiography. For DNase I footprinting using *S. coelicolor* M145 cell extracts, the binding conditions were identical to that of the gel mobility shift assays, and the DNase I digestion was performed as described previously (Kelemen *et al.*, 2001). The dideoxy-sequencing ladder (Amersham Pharmacia biotech; T7 sequencing™ kit) was produced using the same labelled oligonucleotide used to generate the *sspA* probe. DNase I footprinting in the presence of BldD purified from *E. coli* was carried out using single end-labelled *sspA* probes generated by PCR with the primers 7434_F1 and 7434_R1, as described in den Hengst *et al.* (2010).

### Microscopy

For fluorescence microscopy, spores were inoculated alongside microscope coverslips inserted at 45° angles into SFM medium and incubated at 30°C for 48–96 h. Samples were stained with WGA-Alexa488 (Molecular Probes, 50 μg ml^−1^) and/or propidium iodide (Sigma, 25 μg ml^−1^) as described previously (Holmes *et al.*, 2013) and were viewed using Leica TCS SP2 laser-scanning confocal microscope with a 63×, 1.4 NA oil-immersion objective. Scanning electron microscopy and transmission electron microscopy were performed as described previously (Holmes *et al.*, 2013).
